# Comment on Alkouri et al. Metabolic Syndrome in Middle Eastern Patients with Atherosclerotic Cardiovascular Disease: A High Burden Driven by Cumulative Risk Factors. *J. Cardiovasc. Dev. Dis.* 2026, *13*, 240

**DOI:** 10.3390/jcdd13090453

**Published:** 2026-09-10

**Authors:** Yousef Saleh Khader

**Affiliations:** Department of Public Health, Faculty of Medicine, Jordan University of Science and Technology, Irbid 22110, Jordan; yskhader@just.edu.jo; Tel.: +962-79-680-2040

**Keywords:** metabolic syndrome, SMuRFs, atherosclerotic cardiovascular disease, circularity bias, table 2 fallacy, observational epidemiology, Middle East

## Abstract

I read with interest the recent study by Alkouri et al. reporting a high prevalence of metabolic syndrome (MS) among Middle Eastern patients with atherosclerotic cardiovascular disease (ASCVD) and a graded increase in MS prevalence with the cumulative burden of standard modifiable risk factors (SMuRFs). The clinical question is important and the assembled cohort is sizeable. However, several methodological features warrant consideration when interpreting the conclusions and, in my view, merit comment. First, the exposure (SMuRF count), the regression predictors, and the outcome (MS) share constituent components, most directly hypertension and the lipid abnormalities, so that the central graded relationship and the interpretation of several “independent predictors” are partly influenced by definitional overlap. Second, the operational MS definition was modified because of limitations in data availability (fasting glucose omitted, body mass index substituted for waist circumference, and a hypertension threshold of ≥140/90 mmHg), which limits direct comparability of the reported 42.7% prevalence with studies using standard definitions. Third, the pooling of one prospective cohort with six heterogeneous retrospective registries, together with an unexplained reduction from a stated 5540 participants to 1016 analyzed, may introduce selection concerns and limits the extent to which the findings are regionally representative of the Middle East. Fourth, several aspects of the statistical reporting would benefit from clarification. I outline these issues and suggest analyses that would strengthen interpretation.

## 1. Introduction

Alkouri et al. [1] address a genuinely under-studied question, the burden and determinants of MS among Middle Eastern patients with established ASCVD, and assemble a pooled cohort of 1016 patients drawn from the prospective Jordan SMuRF-less Study and six regional cardiovascular registries. They report an overall MS prevalence of 42.7% and a striking graded increase across SMuRF strata (2.2%, 28.3%, and 62.2% for 0, 1–2, and 3–4 SMuRFs, respectively; *p* < 0.001), together with a multivariable model identifying age, diabetes, hypertension, chronic kidney disease, heart failure, body mass index (BMI), and triglycerides as independent predictors of MS [1].

I commend the authors for tackling a regionally relevant problem and for acknowledging several limitations candidly. My concern is that the interpretation of the principal findings, as framed in the title and conclusions, should account for relationships that are partly built into the study’s definitions and for an adopted MS construct that differs from the standard definitions used to contextualize the results. I address four points below, in descending order of importance, and close with constructive suggestions.

## 2. The Exposure, the Predictors, and the Outcome Share Components

The SMuRFs are defined as dyslipidemia, hypertension, diabetes mellitus, and smoking, while MS is defined as the presence of at least three of: hypertension, obesity, low high-density lipoprotein cholesterol (HDL-C), and elevated triglycerides [1]. Hypertension therefore appears in both the exposure count and the outcome definition, and “dyslipidemia”, operationalized partly through lipid values and lipid-lowering therapy, overlaps mechanically with the HDL-C and triglyceride criteria of MS.

As a consequence, the reported graded increase in MS across SMuRF strata may, in part, reflect arithmetic overlap between the definitions rather than an entirely independent empirical association. A SMuRF-less patient is by construction free of hypertension and dyslipidemia and can satisfy MS only through obesity together with the lipid criteria, yet qualifying lipid values would generally trigger the dyslipidemia SMuRF. It is therefore structurally difficult for a SMuRF-less patient to meet MS criteria, which is reflected in the single MS case among 45 such patients (2.2%). The authors note this overlap in their Limitations, and this acknowledgment is important. In my view, the overlap also warrants caution in interpreting causal wording in the title (“…A High Burden Driven by Cumulative Risk Factors”) and in presenting the gradient as an independent substantive finding.

A related concern applies to the interpretation of the logistic regression coefficients. Hypertension, BMI, triglycerides, and HDL-C are entered as predictors of MS, but these are the diagnostic components of MS. An odds ratio for “hypertension predicting MS” (OR 3.07) requires particular caution because hypertension is one of the four criteria that define the outcome; this definitional relationship can mechanically strengthen the observed association. This is a recognized hazard in the interpretation of multivariable coefficients [2], and the authors themselves flag “circularity bias.” I would therefore interpret the coefficients for constituent variables cautiously and avoid presenting them as independent etiologic determinants of MS. This concern relates to the interpretation of these constitutive variables rather than to the overall validity of the regression approach or the remaining statistical analysis.

## 3. A Non-Standard MS Definition Limits Comparability

The authors describe their criteria as a “modified NCEP ATP III” framework. These modifications reflect acknowledged limitations in the availability of variables across the pooled datasets, but they are consequential for comparison with standard validated definitions [3,4,5]:

1. Fasting glucose is omitted entirely. Dysglycemia is one of the five canonical MS criteria; its absence, which the authors acknowledge as a data-availability constraint, means that the operational definition differs from NCEP ATP III, the IDF, and the 2009 harmonized statement [3,4,5].
2. Obesity is operationalized as BMI ≥ 30 kg/m^2^ rather than waist circumference. BMI is an imperfect surrogate for central/visceral adiposity, and cardiometabolic risk in many regional populations manifests at lower BMI thresholds; this may affect classification of the obesity criterion in the population of interest.
3. The hypertension criterion uses ≥140/90 mmHg, whereas the standard MS blood-pressure threshold is ≥130/85 mmHg. The higher cut-point would be expected to reduce the number of participants meeting that criterion relative to standard MS definitions.

The net effect is a modified “≥3 of 4” operational definition (versus the standard ≥ 3 of 5) that is not directly equivalent to published MS criteria. This distinction is important when the Discussion benchmarks the 42.7% prevalence and the male predominance against Western and regional MS literature, because the underlying definitions differ. At minimum, these definitional differences should be stated explicitly wherever cross-study comparisons are drawn, and such comparisons should be interpreted with caution.

## 4. Pooling Heterogeneity, Participant Flow, and the “Middle Eastern” Label

The analytic cohort combines a prospective ASCVD cohort with six retrospective registries that differ substantially in design and case-mix, including a percutaneous coronary intervention registry, an atrial fibrillation registry, an acute myocardial infarction/statin-eligibility registry, and a women-only registry restricted to ages 18–45 [1]. These sources differ in age, sex, and indication in ways that directly shape the very distributions being interpreted; for example, the finding that MS is more prevalent in men is sensitive to which registries contributed which sexes. However, the analyses appear to treat the pooled sample as a single cohort, without a random-effects framework, a study-source covariate, an assessment of between-source heterogeneity, or sensitivity analyses excluding individual registries.

The participant flow is also difficult to reconcile. The Informed Consent Statement refers to “a combined database of 5540 participants” [1], whereas the analytic sample is 1016; the basis for this substantial reduction is not fully described, and no flow diagram is provided. Moreover, the registry sizes reported in Table 1 sum to substantially more than 5540 before all sources are counted, so the numbers as presented do not add up. A STROBE-compliant flow diagram [6] reconciling source registries to the combined database to the analytic sample, with reasons for exclusion, would allow readers to better assess the potential for selection bias.

Finally, the authors note that country-level identifiers were not uniformly available, and the pooled sample appears to be predominantly derived from Jordanian cohorts. The “Middle Eastern” framing of the title and Conclusions, and the regionally specific interpretations (e.g., sociocultural explanations for the male predominance), should therefore be understood as having limited regional representativeness rather than as applying uniformly across the Middle East.

## 5. Statistical Reporting

Several statements warrant clarification:

- Normality. The Methods report that Shapiro–Wilk testing showed “*p*-values greater than 0.05” for all continuous variables, indicating normal distributions. This would benefit from clarification, particularly because the preceding sentence specifies non-parametric tests for non-normally distributed variables. It would be helpful to describe how distributional assumptions were assessed and how the choice between parametric and non-parametric tests was made [7].
- Dispersion. The non-MS HDL-C value is reported as 46.4 ± 25.5 mg/dL. Because the mean and standard deviation alone do not characterize the shape of the distribution, additional distributional summaries would help readers interpret this variability.
- Model diagnostics. The logistic regression is reported without the number of candidate variables, multicollinearity diagnostics (particularly relevant given the overlapping predictors in Section 2), goodness-of-fit (e.g., Hosmer–Lemeshow), or discrimination (c-statistic). Notably, the SMuRF burden, the study’s central exposure, does not appear in the model; its individual components are entered instead.
- Treatment effects on the outcome. MS is ascertained in a heavily treated secondary-prevention population (statins 88%), and the authors invoke a statin effect to explain lower LDL-C in the MS group. The same consideration may also affect lipid-based MS criteria (HDL-C, triglycerides) and therefore merits acknowledgment when interpreting MS classification and lipid associations.
- Minor inconsistencies. Tables 3 and 4 are titled N = 1059, but the cell counts sum to 1016 (434 + 582). One-year survival is listed among collected variables but is not analyzed.

## 6. Conclusions and Suggestions

The clinical question posed by Alkouri et al. is worthwhile, and the assembled dataset is a regional asset. My concern is primarily interpretive: the headline gradient and some “independent predictors” are influenced by definitional overlap, the modified MS construct limits direct comparison with standard definitions, and the pooled, heavily attrited sample limits the degree of regional generalization. We respectfully suggest that the findings be read primarily as descriptive of the studied secondary-prevention population, with caution in interpreting cumulative risk factors as independently “driving” MS.

Several steps would strengthen the work or any future analysis of these data:

1. Reframe the SMuRF–MS relationship as primarily descriptive, and avoid interpreting constituent components of MS (hypertension, BMI, triglycerides, HDL-C) as independent etiologic predictors of the syndrome. For models intended to identify determinants beyond the definition itself, consider focusing on variables external to the MS definition.
2. State explicitly, wherever comparisons are made, that because of data-availability limitations the adopted MS definition omits glucose, substitutes BMI for waist circumference, and uses a ≥140/90 mmHg threshold; interpret comparisons with studies using standard definitions cautiously, and consider sensitivity analyses against a standard (e.g., 2009 harmonized) definition where data permit [5].
3. Provide a STROBE flow diagram [6] and add a study-source term (or stratified/sensitivity analyses by registry) to address pooling heterogeneity; temper region-wide generalizations to reflect the actual composition and limited regional representativeness.
4. Clarify the normality assessment and the rationale for choosing parametric versus non-parametric tests for continuous variables, and report standard model-fit and collinearity diagnostics where appropriate.

I thank the authors for a stimulating contribution and hope these observations are received in the constructive spirit intended.

## Data Availability

No new data were created or analyzed in this study. Data sharing is not applicable to this article.
